# Dietitians’ practices in dialysis units in Brazil: nutritional assessment and intervention

**DOI:** 10.1590/2175-8239-JBN-2023-0092en

**Published:** 2024-02-09

**Authors:** Fabiana Baggio Nerbass, Aline de Araujo Antunes, Lilian Cuppari

**Affiliations:** 1 Fundação Pró-Rim, Joinville, SC, Brazil.; 2 Comitê de Nutrição da Sociedade Brasileira de Nefrologia, São Paulo, SP, Brazil.; 3 Universidade Federal de São Paulo, São Paulo, SP, Brazil

**Keywords:** Dietitians, Dialysis, Nutritional Assessment

## Abstract

**Introduction:**

The importance of dietitians in dialysis units is indisputable and mandatory in Brazil, but little is known about the practices adopted by these professionals.

**Objective:**

To know practices adopted in routine nutritional care, focusing on nutritional assessment tools and treatment strategies for people at risk or diagnosed with malnutrition.

**Methodology:**

Electronic questionnaire disseminated on social media and messaging applications. It included questions that covered dietitians’ demographic and occupational profile characteristics and of the dialysis unit, use and frequency of nutritional assessment tools, nutritional intervention strategies in cases of risk or diagnosis of malnutrition, prescription and access to oral supplements.

**Results:**

Twenty four percent of the Brazilian dialysis units (n = 207) responded electronically. The most used nutritional assessment tools with or without a pre-established frequency were dietary surveys (96%) and Subjective Global Assessment (83%). The strategies in cases of risk or presence of malnutrition used most frequently (almost always/always) were instructions to increase energy and protein intake from foods (97%), and increasing the frequency of visits (88%). The frequency of prescribing commercial supplements with standard and specialized formulas was quite similar. The availability of dietary supplements by the public healthcare system to patients varied between regions.

**Conclusion:**

Most dietitians use various nutritional assessment tools and intervention strategies in cases of risk or malnutrition; however, the frequency of use of such tools and strategies varied substantially.

## Introduction

The nutritional status assessment of patients undergoing dialysis therapy is of great importance for appropriate intervention and detection of those with malnutrition or at risk of malnutrition, factors that are known to affect the prognosis of these individuals. There are several tools dietitians’ can use for this purpose; although there is still no sufficient evidence to indicate the superiority of any. Therefore, the dietitian should carry out a comprehensive nutritional assessment, including assessment of appetite, food consumption, anthropometric and body composition measurements, biochemical data, and physical examination with a nutritional focus^1^.

The importance of the dietitian’s role in dialysis units is indisputable and mandatory in Brazil (RDC nº 154 of the Ministry of Health, of June 15, 2004)^2^, but little is known about the practices adopted by these professionals. Therefore, an electronic questionnaire was formulated and aimed at dietitians working in dialysis units, seeking to understand the profile and practices of this professional group. In a previous paper, we described the demographic profile of the participants and the clinics they worked. The findings showed a large variation in relation to the number of patients per professional workload and also the percentage of individuals who receive monthly care in the units^3^.

In this article, we present and discuss the results related to the practices adopted in routine nutritional care, focusing on nutritional assessment tools and treatment strategies for people at risk or diagnosed with malnutrition.

## Methods

The questionnaire formulated by members of the Nutrition Committee of the Brazilian Society of Nephrology (BSN) and other leading dietitians in the field of Nephrology was published in September, 2022, through BSN’s social networks and disseminated to professionals in groups of messaging apps. For participation, identification of the professional was optional and identification of the dialysis unit in which they worked was not requested. If the professional worked in more than one unit, they were instructed to repeat the questionnaire.

The questionnaire answered using the Google Forms tool contained questions that covered characteristics of the dialysis units and the demographic and occupational profile of the nutrition professionals, in addition to the frequency of nutritional care provided to patients. In this article, we describe and discuss the results related to the use and frequency of nutritional assessment tools, nutritional intervention strategies in cases of risk or diagnosis of malnutrition, in addition to the prescription and availability of oral dietary supplements. The questions related to this article can be viewed in the supplementary material.

We calculated the total percentage of patients seen monthly by the dietitian. The number of patients (N) in the unit per monthly workload was obtained by dividing the N by the weekly workload multiplied by five. Ex.: In a unit with 120 patients in which the dietitian works for 20 hours per week, the number of patients per hour per month is equal to 1.2 (120/100).

### Statistical Analysis

Statistical analysis was performed using the SPSS software, version 21.0 for Windows (SPSS, Inc. Chicago, IL, USA). The results were presented as percentages, medians and interquartiles, when appropriate. To compare variables between the groups, the chi-square test was used for categorical variables and the Mann-Whitney test for continuous variables. Statistical significance was considered for a value of P < 0.05.

## Results

Two hundred and seven questionnaires were answered electronically, equivalent to 24% of the 849 active dialysis units registered within the BSN at the time^4^, completed by 202 dietitians (one worked in three units and other three, in two different units). The Northeast and Central-West regions were those with the highest percentage of participation units (28%), followed by the South and North (27%) and Southeast (21%). The main characteristics of the participants and the dialysis units in which they worked are shown in Table 1.

**Table 1:** Main characteristics of participating dietitians and dialysis units

Time working in a dialysis unit	
<2 years	27%
3 to 4 years	14%
5 to 10 years	28%
>10 years	30%

Additional education in nephrology	
None	17%
Training	30%
Specialization	43%
MSc and/or PhD	10%

Weekly working hours in the unit	
<20 hours	14%
20 to 30 hours	54%
>30 hours	32%

Activities beyond the dialysis care	
Conservative care	36%
Kidney transplant care	16%
Nutritional management	59%

Funds for the dialysis treatment	
Public only	20%
Mainly public	57%
Private only	15%
Mainly private	8%

The median (interquartiles) of hemodialysis patients per unit was 191 (120 – 262). Of the 207 units, 116 (56%) also offered peritoneal dialysis therapy, and the median number of patients in this modality was 15 (4 – 40). Considering both modalities, the units had 200 (129 – 300) patients on renal replacement therapy.

Regarding the aspects considered in routine nutritional care, 99% of dietitians regularly analyzed laboratory tests and an equal percentage evaluate interdialytic weight gain, 95%, appetite; 94%, signs and symptoms of the gastrointestinal tract; 90% consider eating habits; and 85%, changes in dry weight.

**Figure 1.**
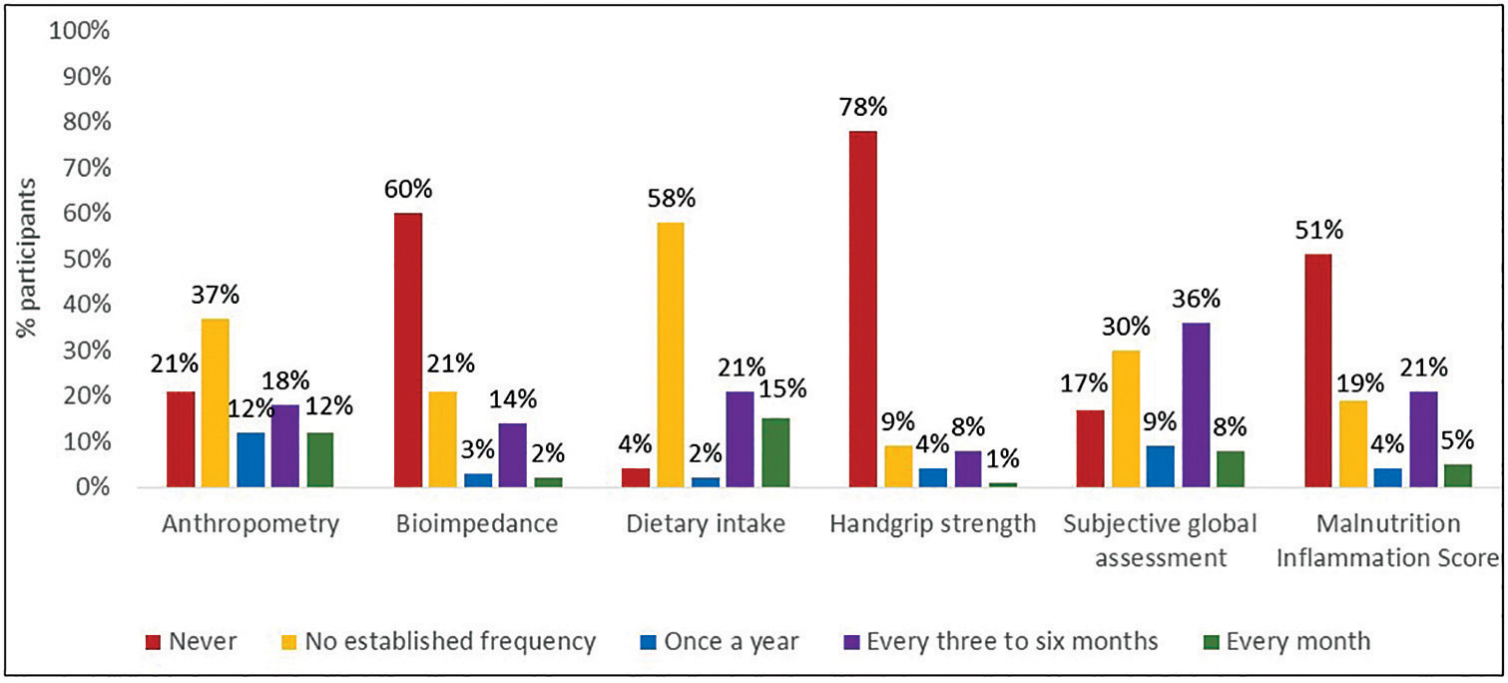
Nutritional assessment tools – Use and frequency of use.

The type and frequency of use of nutritional assessment tool use are shown in Figure 1. Among the six tools in the questionnaire, those with the highest percentage of non-use by participants were handgrip strength (78%), bioimpedance (60%) and Malnutrition Inflammation Score – MIS (51%). The most used, whether with or without established periodicity, were dietary surveys (96%), Subjective Global Assessment – SGA (83%) and body composition by anthropometry (79%).

When comparing the use of tools between units funded mainly by the public and private systems, regardless of frequency, we found a statistical difference only in relation to bioimpedance, being used in 33% of units with public funding and in 64% of those with private funding (P < 0.001).

**Figure 2.**
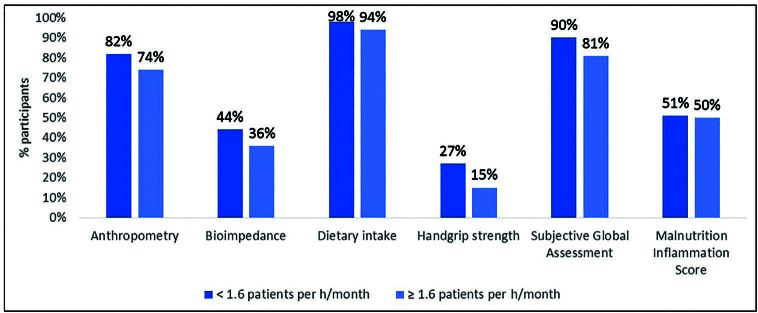
Nutritional assessment tools use, regardless of periodicity; according to the median value of the number of patients in the unit by the dietitian’s workload.

The median (interquartile) number of patients per monthly working hour of the dietitian was 1.6 (1.0 – 2.3). When dividing into two groups according to this median, there was a higher percentage of dietitians with fewer patients per workload using nutritional assessment tools, regardless of frequency, although the difference was small (Figure 2).

**Figure 3.**
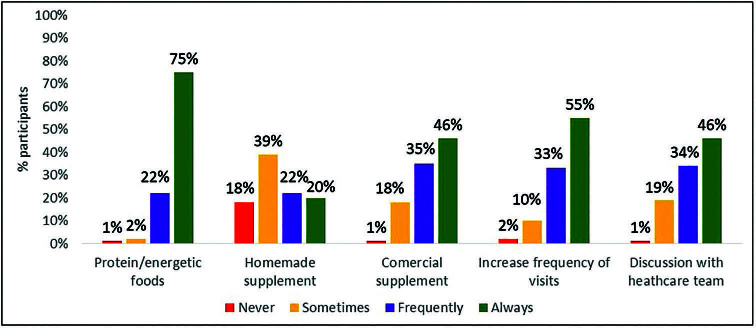
Strategies pointed out by the nutritionies in cases of malnutrition risk of diagnosis thereof.

The strategies in cases of risk or presence of malnutrition used most frequently (always or almost always) by dietitians were education on increasing energy and protein intake from food (97%) and increasing the frequency of visits (88%), followed by the prescription of commercial oral supplements (81%), discussion with the multidisciplinary team (80%) (Figure 3). The least used was the prescription of homemade supplements, with 42% prescribing them almost always or always.

Regarding commercial oral supplements, 7% (N = 14) of the units provided them to some patients to whom it was indicated; 10% (N = 20), to all with indication; and the remaining did not provide it. Among the 34 units that provided supplements, 31 were funded mainly (N = 13) or exclusively by the public healthcare system (N = 18).

For the question “In cases of risk or diagnosis of malnutrition in which commercial supplementation is indicated and the patient does not have the financial resources to purchase it, does the public healthcare service provide it?”, the answers were: never, 17%; almost never, 27%; half of the time, 14%; most of the time, 27%; always, 6%; and not known, 9%. Among the regions, the one that provided supplementation most frequently, between half of the time and always when requested, was the South region (71%), followed by the Northeast (55%), North (50%), Mid-West (49%) and Southeast (40%).

**Figure 4.**
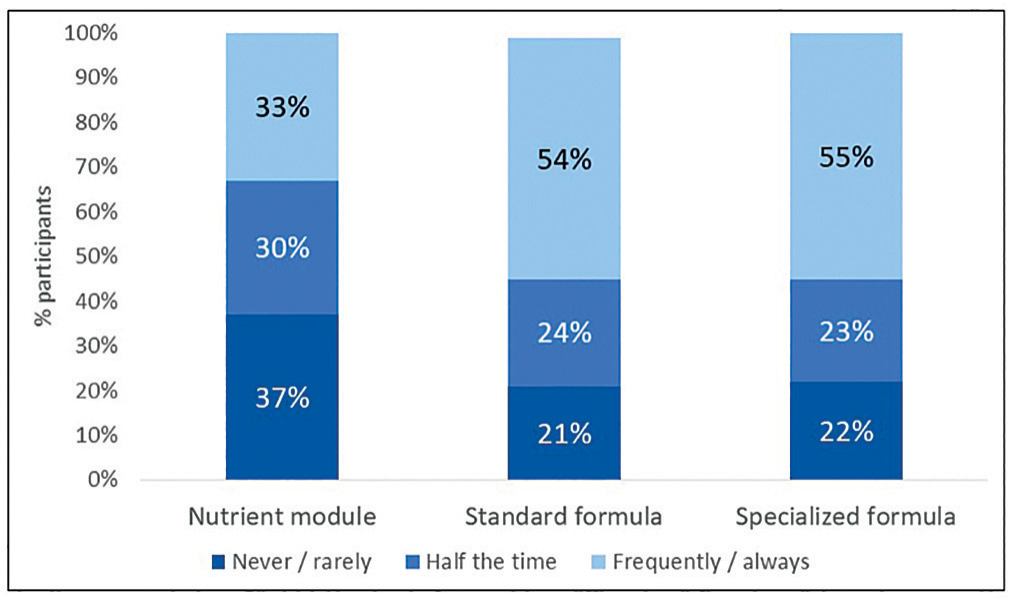
Oral supplement types – frequency of prescription.

Figure 4 shows the answers to the question “Based on your clinical practice in this unit, the purchasing power of your patients and the availability of the public healthcare service, when is it necessary to recommend a commercial supplement, how often do you prescribe standard formulas, specialized formulas and nutrient modules?” The frequency of prescription of standard and specialized formulas was quite similar and more frequent than that of nutrient modules.

## Discussion

In this study, which included the participation of around a quarter of dietitians working in dialysis units in Brazil, we found that most use several nutritional assessment tools and intervention strategies in cases of risk or malnutrition, but the frequency of use of such tools and strategies showed considerable variability.

In routine nutritional care, most participants stated that they regularly analyzed laboratory tests, interdialytic weight gain, appetite, signs and symptoms of the gastrointestinal tract, eating habits and changes in dry weight, which is in accordance with the practices proposed by KDOQI/AND clinical practice guideline for nutrition in CKD)^1^, although it was not possible to identify the frequency with which these analyzes are carried out.

The most used nutritional status assessment tools were anthropometry, SGA and dietary surveys, possibly due to their lower cost and simple application. All are recommended as they provide reliable information regarding the patient’s nutritional condition. Regarding periodicity, the KDOQI/AND guideline suggests that the assessment of nutritional status to be comprehensive and carried out at least within the first 90 days of starting dialysis, annually, or when indicated by nutritional screening or standardization of care^1^. In a previous article with results referring to the same questionnaire, it was reported that 64% of patients are seen monthly by dietitians. However, according to the information collected, it was not possible to identify which activities are specifically carried out during monthly care, whether only based on clinical symptoms and routine examinations or whether they included a more comprehensive nutritional assessment.

Regarding the assessment of patients’ body composition, in 40% of units it is carried out using electrical bioimpedance. It is worth noting that the majority of units that have bioimpedance equipment available are those maintained with private funds, which can be explained by the high cost, especially the multifrequency model, suggested by the KDOQI/AND guideline as the most suitable for this population^1^. Another little used tool was the dynamometer for measuring handgrip strength. This measure has gained importance due to its relationship with muscle mass and functionality, and it has been suggested to be used as the main marker for identifying patients with sarcopenia^5^. This tool is also recommended by the guidelines as a marker of nutritional and functional status^1^. Although not evaluated, it is possible to assume that its little use by dietitians is also related to cost.

Dietitians with a lower number of patients per hour per month use nutritional assessment tools more frequently, although with a small difference in relation to the others. In fact, in a survey carried out with dietitians who work in dialysis units in the USA, the high number of patients per professional was pointed out by 40% of the 951 participants as a barrier to implementing practicies suggested by the guidelines^6^.

It is worth noting that the time spent on other activities in dialysis units, such as managing food services and caring for patients undergoing conservative treatment, was not in the questionnaire, making it impossible to assess the influence of this activity on the characteristics and quality of clinical care for patients on dialysis. A North American study that included 14 dietitians in a time and movement study found that, on average, only 25% of the time was dedicated to direct patient care. Indirect care, which includes time spent on care plans, checking and noting medical records, communicating with other professionals, among others, occupied 56% of the time^7^. As evidenced in another investigation with the participation of 466 dietitians, administrative activities reduce the time available for interaction with patients, so necessary to improve nutritional outcomes^8^.

One of the strategies used by almost all dietitians, in the presence of risk or diagnosis of malnutrition, was the provision of dietary education aimed at increasing food intake to provide a greater supply of energy and protein. This is the recommendation indicated by the guidelines as an initial approach to nutritional care. If this does not produce the desired effects, oral supplementation is then recommended as a valid strategy. In the present study, 81% of dietitians reported prescribing commercial oral supplements for patients with this condition.

Oral supplementation can provide an additional energy supply of around 7–10 Kcal/Kg/day and of 0.3–0.4 g/Kg/day of protein, requiring a minimum spontaneous intake of 20 Kcal/Kg and 0.4–0.8 g of protein/kg in order to meet the recommendations for daily energy and protein intake^9^. Studies show that the use of nutritional supplements, especially during hemodialysis sessions, minimizes its catabolic effect, which can contribute to improving nutritional status. In an investigation with malnourished patients, oral supplementation over six months led to a 14% increase in the SGA score, in addition to an increase in albumin and pre-albumin concentrations^10^. Although there is evidence of benefits from this practice, the data show that few clinics (n = 34) provide supplementation to their patients; and, surprisingly 31 of them are partially or fully funded by the public healthcare system. We speculate that such clinics may be linked to hospital units, which would facilitate the provision of supplements to patients undergoing dialysis treatment.

Regarding the participation of the public healthcare system in providing free supplementation to patients with financial limitations, there is a discrepancy among the regions of the country, with the southern region of the country being the most supportive. On the other hand, the Southeast region provides supplementation free of charge to only 40% of patients who require it.

The specific nutritional supplements for people with chronic kidney disease on dialysis available in our country, have hypercaloric (≥1.5 Kcal/mL) and high protein (>13 g/200 mL) characteristics. When compared to standard hypercaloric and hyper protein supplements, they have a lower sodium and potassium content, with a variable phosphorus content, depending on the product. On the other hand, when comparing the price of these supplements, specific supplements cost around 50% more than standard supplements. Thus, when indicating the type of supplementation to the patient (specific or standard), the dietitian must individually consider the patient’s clinical and biochemical aspects, as well as the financial viability so that treatment can be maintained for the indicated period, when there is no provision by the public healthcare system.

As a limitation, we highlight the possibility that the findings do not faithfully reflect the national reality, since this study was disseminated through a previously organized professional messaging application, in addition to BSN’s social networks. As strengths, we highlight the originality and the participation of approximately a quarter of professionals working in dialysis units in the country.

With the information collected in this study, we report the diversity in the practice of dialysis dietitians in Brazil in relation to nutritional assessment and intervention in cases of risk of or malnutrition. Among other causes, this diversity reflects the lack of regulation of this professional’s work, with the establishment of a minimum workload based on the number of patients and the duties performed by the professional. The establishment of adequate parameters and routines for nutritional assessment and intervention is relevant to improving the quality of care and the lives of these patients.

## Supplemental Material

The following online material is available for this paper:

Questionnaire extracted from Google Forms: DIETITIANS’ PROFILE AND PRACTICES IN BRAZILIAN DIALYSIS UNITS
