# Supplementary material for: Dietitians’ practices in dialysis units in Brazil: nutritional assessment and intervention
Source: J Bras Nefrol. 2024 Feb 9;46(3):e20230092. doi: 10.1590/2175-8239-JBN-2023-0092en (PMC11296690; doi:10.1590/2175-8239-JBN-2023-0092en)
Supplement: Supplementary file 1 [file 2175-8239-jbn-46-3-e20230092-en-s1.pdf]

Braz. J. Nephrol. (J. Bras. Nefrol.)
https://doi.org/10.1590/2175-8239-JBN-2023-0092en

Supplement Material for “Dietitians´ practices in dialysis units in Brazil: nutritional assessment and intervention”

Questionnaire extracted from Google Forms.

DIETITIANS´ PROFILE AND PRACTICES IN BRAZILIAN DIALYSIS UNITS

Dear fellow,

With the aim of understanding the profile and the practices of dietitians working in dialysis units in our country, the Nutrition Committee of the Brazilian Society of Nephrology (BSN) formulated a simple and objective questionnaire. Your identification and of the clinic where you work are not necessary. If you work in more than one dialysis unit, you can answer more than once. The estimated time to participate is just 5 minutes.

We really appreciate your participation.

BSN Nutrition Committee

Name (optional):

Email (optional):

Do you have additional education in the field of Nephrology?

( ) No

( ) Yes, training

( ) Yes, specialization

( ) Yes, master's degree and/or doctorate

Other:

How long have you worked in a dialysis unit(s)?

( ) Less than a year

( ) 1 to 2 years

( ) 3 to 4 years

( ) 5 to 10 years

( ) 10 to 20 years

( ) More than 20 years

About the dialysis unit where you currently work: in which Brazilian state is it located?

What is the payment source(s) for the dialysis unit?

( ) Public funds only.

( ) Private funds only.

( ) Predominantly public.

( ) Predominantly private agreements.

Other:

What is your weekly working hours at the unit?

On average, how many hemodialysis patients does the unit have?

Regarding peritoneal dialysis (PD):

Does the unit have PD patients? ( ) Yes No ( )

Are you responsible for the nutritional care of these patients? (   ) Yes No ( )

If yes, on average, how many patients are on peritoneal dialysis?

On average, how many patients in the dialysis unit (HD and/or PD) receive nutritional care in a month?

In routine nutritional care, which nutritional aspects are typically addressed?

( ) Appetite.

( ) Interdialytic weight gain and/or fluid overload.

( ) Changes in dry weight.

( ) Laboratory tests.

( ) Eating habit.

( ) Gastrointestinal signs and symptoms.

Other:

In addition to routine nutritional care, are the nutritional assessment tools listed below used? If yes, how often?

	Never use	Use sporadically, without an established schedule	Every month	Every three months	Every six months	Once a year
Body composition by anthropometry						
Body composition by bioimpedance						
Dietary surveys (records, food frequency)						
Subjective Global Assessment						
Handgrip strength						
MIS (Malnutrition Inflammation Score)						

In cases of risk or diagnosis of malnutrition, what treatment strategies are used?

	Never	Sometimes	Almost always	Always
Increase frequency of visits				
Discussion with healthcare team				
Potein / energetic foods recommendations				
Homemade supplements				
Commercial supplements				

Does the clinic you work at provide free commercial supplements to dialysis patients?

( ) No.

( ) Yes, for everyone who needs it.

( ) Yes, for some of those who need it.

Other:

In cases of risk or diagnosis of malnutrition in which commercial nutritional supplementation is indicated and the patient does not have the financial resources to purchase it, does the public healthcare service provide it?

( ) I have no knowledge.

( ) Sometimes.

( ) Most of the time.

( ) Half the time.

( ) Almost never.

( ) Never.

Other:

Based on your clinical practice in this unit, the purchasing power of your patients and the availability of the public healthcare service, when it is necessary to recommend a commercial supplement, how often do you prescribe standard formulas, specialized formulas and nutrient modules?

	Always	Most of the times	Half of the times	Almost never	Never
Standard formula					
Specialized formula (CKD, diabetes, etc.)					
Modules (carbohydrates, proteins)					
