# Supplementary material for: Dietitians’ practices in dialysis units in Brazil: nutritional assessment and intervention
Source: J Bras Nefrol. 2024 Feb 9;46(3):e20230092. doi: 10.1590/2175-8239-JBN-2023-0092en (PMC11296690; doi:10.1590/2175-8239-JBN-2023-0092en)
Supplement: Supplementary file 2 [file 2175-8239-jbn-46-3-e20230092-pt-s1.pdf]

Braz. J. Nephrol. (J. Bras. Nefrol.)
https://doi.org/10.1590/2175-8239-JBN-2023-0092en

Material Suplementar para "Práticas de nutricionistas em unidades de diálise no Brasil: avaliação e intervenção nutricionais"

Questionário extraído do *Google Forms.*

PERFIL E PRÁTICAS DO NUTRICIONISTA EM UNIDADES DE DIÁLISE BRASILEIRAS

Prezado(a) colega nutricionista,

Com o objetivo de conhecer o perfil e algumas práticas de nutricionistas atuantes em unidades de diálise em nosso país, o Comitê de Nutrição da Sociedade Brasileira de Nefrologia (SBN) formulou um questionário simples e objetivo. Sua identificação e da clínica em que atua não são necessárias. Caso trabalhe em mais de uma unidade de diálise, você pode responder outras vezes.
O tempo estimado para participar é de apenas 5 minutos.
Agradecemos muito a sua participação.
Comitê de Nutrição da SBN

Nome (opcional):

E-mail (opcional):

Possui formação complementar na área de Nefrologia?

( ) Não

( ) Sim, capacitação

( ) Sim, especialização

( ) Sim, mestrado e/ou doutorado

Outro:

Há quanto tempo atua em unidade(s) de diálise?

( ) Menos de um ano

( ) 1 a 2 anos

( ) 3 a 4 anos

( ) 5 a 10 anos

( ) 10 a 20 anos

( ) Mais de 20 anos

Sobre a unidade de diálise em que você atua atualmente: em qual estado brasileiro ela está localizada?

Qual(is) a(s) fonte(s) pagadora(s) da unidade de diálise?

( ) Apenas SUS.

( ) Apenas convênios privados.

( ) Predominantemente SUS.

( ) Predominantemente convênios privados.

Outro:

Qual a sua carga horária semanal de trabalho na unidade?

A unidade possui em média quantos pacientes em hemodiálise?

Com relação à diálise peritoneal (DP):

A unidade possui pacientes em DP? (   ) Sim (   ) Não

Você é responsável pelo atendimento nutricional desses pacientes? (   ) Sim (   ) Não

Se sim, possui em média quantos pacientes em diálise peritoneal?

Em média, quantos pacientes da unidade de diálise (HD e/ou DP) recebem atendimento nutricional em um mês?

No atendimento nutricional de rotina, quais aspectos nutricionais são normalmente abordados?

( ) Apetite.

( ) Ganho de peso interdialítico e/ou sobrecarga hídrica.

( ) Mudanças no peso seco.

( ) Exames laboratoriais.

( ) Hábito alimentar.

( ) Sinais e sintomas gastrointestinais.

Outro:

Além do atendimento nutricional de rotina, as ferramentas de avaliação nutricional indicadas abaixo são utilizadas? Se sim, com qual frequência?

	Nunca utilizo	Utilizo esporadicamente, sem periodicidade estabelecida	Todos os meses	A cada três meses	A cada seis meses	Uma vez ao ano
Composição corporal por antropometria						
Composição corporal por bioimpedância						
Inquérito dietético (recordatórios, registros, frequência alimentar)						
Avaliação subjetiva global						
Força de preensão manual						
MIS (Malnutrition Inflammation Score)						

Em casos de risco ou diagnóstico de desnutrição, quais estratégias de tratamento são utilizadas?

	Nunca	Às vezes	Quase sempre	Sempre
Aumento da periodicidade de atendimento				
Discussão do caso com equipe multidisciplinar				
Recomendações sobre alimentos energéticos, proteicos				
Suplemento alimentar caseiro				
Suplemento alimentar industrializado				

A clínica em que você trabalha fornece gratuitamente suplemento industrializado aos pacientes em diálise?

( ) Não.

( ) Sim, para todos que necessitam.

( ) Sim, para parte dos que necessitam.

Outro:

Em casos de risco ou diagnóstico de desnutrição em que há indicação de suplementação nutricional industrializada e o paciente não possui recursos financeiros para a aquisição, o serviço público de saúde fornece?

( ) Não tenho conhecimento.

( ) Sempre.

( ) A maioria das vezes.

( ) Metade das vezes.

( ) Quase nunca.

( ) Nunca.

Outro:

Com base na sua prática clínica nesta unidade, o poder aquisitivo dos seus pacientes e a disponibilidade do serviço público de saúde, quando é necessária a indicação de um suplemento alimentar industrializado, com qual frequência você prescreve fórmulas padrão, fórmulas especializadas e módulos de nutrientes?

	Sempre	A maioria das vezes	Metade das vezes	Quase nunca	Nunca
Fórmula padrão					
Fórmula especializada (DRC, diabetes, etc.)					
Módulos (carboidratos, proteínas)					
