# Supplementary material for: Dietitians’ practices in dialysis units in Brazil: nutritional assessment and intervention
Source: J Bras Nefrol. 2024 Feb 9;46(3):e20230092. doi: 10.1590/2175-8239-JBN-2023-0092en (PMC11296690; doi:10.1590/2175-8239-JBN-2023-0092en)
Supplement: Translation

# Translation: Práticas de nutricionistas em unidades de diálise no Brasil: avaliação e intervenção nutricionais

**DOI:** 10.1590/2175-8239-JBN-2023-0092pt

**Authors:** Fabiana Baggio Nerbass, Aline de Araujo Antunes, Lilian Cuppari

**Affiliations:** 1 Fundação Pró-Rim, Joinville, SC, Brasil.; 2 Comitê de Nutrição da Sociedade Brasileira de Nefrologia, São Paulo, SP, Brasil.; 3 Universidade Federal de São Paulo, São Paulo, SP, Brasil.

**Keywords:** Nutricionistas, Diálise, Avaliação Nutricional

## Abstract

**Introdução:**

A importância da atuação do nutricionista em unidades de diálise é indiscutível e obrigatória no Brasil, porém pouco sabemos sobre as práticas adotadas por esses profissionais.

**Objetivo:**

Conhecer práticas adotadas na rotina dos atendimentos nutricionais, com foco nas ferramentas de avaliação nutricional e nas estratégias de tratamento das pessoas com risco ou diagnóstico de desnutrição.

**Metodologia:**

Questionário eletrônico divulgado em mídias sociais e aplicativos de mensagens. Incluiu questões que abrangiam características do perfil demográfico e ocupacional do profissional e da unidade de diálise, utilização e frequência de ferramentas de avaliação nutricional, estratégias de intervenção nutricional em casos de risco ou diagnóstico de desnutrição e prescrição e acesso a suplementos alimentares orais.

**Resultados:**

Foram recebidos eletronicamente o equivalente a 24% das unidades de diálise brasileiras (n = 207). As ferramentas de avaliação nutricional mais utilizadas com ou sem frequência pré-estabelecida foram inquéritos dietéticos (96%) e Avaliação Global Subjetiva (83%). As estratégias em casos de risco ou presença de desnutrição utilizadas com mais frequência (quase sempre/sempre) foram a orientação de incremento energético e proteico por meio de alimentos (97%) e o aumento da periodicidade das visitas (88%). A frequência de prescrição de suplemento industrializado de fórmula padrão e especializada foi bastante semelhante. A disponibilização de suplementos alimentares pelo Sistema Único de Saúde aos pacientes variou entre as regiões.

**Conclusão:**

A maior parte dos nutricionistas utiliza diversas ferramentas de avaliação nutricional e estratégias de intervenção em casos de risco ou desnutrição, porém a frequência de utilização de tais ferramentas e estratégias foi bastante variada.

## Introdução

A avaliação do estado nutricional de pacientes em terapia dialítica é de grande importância para intervenção apropriada e detecção daqueles com desnutrição ou em risco de desnutrição, fatores que sabidamente afetam o prognóstico desses indivíduos. Para essa tarefa, diversas ferramentas podem ser utilizadas pelo nutricionista, ainda sem evidências suficientes que apontem a superioridade de alguma. Assim, é recomendado que o nutricionista realize uma avaliação nutricional abrangente, incluindo a avalição de apetite, consumo alimentar, medidas antropométricas e de composição corporal, dados bioquímicos, e exame físico com foco nutricional^1^.

A importância da atuação do nutricionista em unidades de diálise é indiscutível e obrigatória no Brasil (RDC nº 154 do Ministério da Saúde, de 15 de junho de 2004)^2^, porém pouco sabemos sobre as práticas adotadas por esses profissionais. Diante disso, foi formulado um questionário eletrônico direcionado aos nutricionistas atuantes em unidades de diálise, buscando conhecer o perfil e as práticas desse grupo profissional. Em artigo anterior, descrevemos o perfil demográfico dos participantes e das clínicas que atuam. Os achados evidenciaram uma grande variação em relação ao número de pacientes por carga horária do profissional e também do percentual de indivíduos que recebem atendimento mensal entre as unidades^3^.

Neste artigo, apresentamos e discutimos os resultados encontrados em relação às práticas adotadas na rotina dos atendimentos nutricionais, com foco nas ferramentas de avaliação nutricional e nas estratégias de tratamento das pessoas com risco ou diagnóstico de desnutrição.

## Métodos

O questionário formulado por membros do Comitê de Nutrição da Sociedade Brasileira de Nefrologia (SBN) e por outros nutricionistas de referência na área da Nefrologia foi divulgado durante o mês de setembro de 2022 por meio das redes sociais da SBN e disseminado para profissionais em grupos de aplicativos de mensagens. Para participação, a identificação do profissional era opcional e não foi solicitada a identificação da unidade de diálise de atuação. Caso o profissional atuasse em mais de uma unidade, foi orientado a repetir o preenchimento.

O questionário respondido por meio da ferramenta *Google Forms* continha questões que abrangiam características das unidades de diálise e do perfil demográfico e ocupacional dos profissionais, além da periodicidade de atendimento nutricional aos pacientes. Neste artigo, descrevemos e discutimos os resultados relacionados à utilização e frequência de ferramentas de avaliação nutricional, estratégias de intervenção nutricional em casos de risco ou diagnóstico de desnutrição, além da prescrição e disponibilidade de suplementos alimentares orais. As perguntas do questionário relacionadas a este artigo podem ser visualizadas no material suplementar.

Calculamos o percentual total de pacientes atendidos mensalmente pelo nutricionista. O número de pacientes (N) da unidade por carga horária mensal foi obtido dividindo o N pela carga horária semanal multiplicada por cinco. Ex.: Em uma unidade com 120 pacientes na qual o nutricionista atua por 20 horas semanais, a quantidade de pacientes por hora mensal é igual a 1,2 (120/100).

### Análise Estatística

A análise estatística foi realizada com o software SPSS, versão 21.0 para Windows (SPSS, Inc. Chicago, IL, EUA). Os resultados foram apresentados em percentual, mediana e interquartis, quando apropriado. Para comparação das variáveis entre os grupos, foi utilizado o teste qui-quadrado para as categóricas e Mann-Whitney para as variáveis contínuas. A significância estatística foi considerada para valor de P < 0,05.

## Resultados

Foram respondidos eletronicamente 207 questionários, o equivalente a 24% das 849 unidades de diálise ativas cadastradas na SBN na época^4^, preenchidos por 202 nutricionistas (um atuava em três unidades e outros três, em duas unidades distintas). As regiões Nordeste e Centro-Oeste foram as com maior percentual de participação de unidades (28%), seguidas pela Sul e Norte (27%) e Sudeste (21%). As principais características dos participantes e das unidades de diálise em que atuavam estão na Tabela 1.

**Tabela 1:** Principais características dos nutricionistas e das unidades de diálise participantes.

Tempo de atuação em unidades de diálise	
<2 anos	27%
3 a 4 anos	14%
5 a 10 anos	28%
>10 anos	30%

Formação complementar em nefrologia	
Nenhuma	17%
Capacitação	30%
Especialização	43%
Mestrado e/ou doutorado	10%

Carga horária semanal na unidade	
<20 horas	14%
20 a 30 horas	54%
>30 horas	32%

Atividades além do atendimento da diálise	
Atendimento conservador	36%
Atendimento transplante renal	16%
Gestão serviço alimentação	59%

Fonte pagadora do tratamento dialítico	
Exclusivamente público	20%
Predominantemente público	57%
Exclusivamente privado	15%
Predominantemente privado	8%

A mediana (interquartis) de pacientes em hemodiálise por unidade foi de 191 (120 – 262). Das 207 unidades, 116 (56%) também ofereciam terapia de diálise peritoneal, e a mediana de pacientes nessa modalidade era de 15 (4 – 40). Considerando as duas modalidades, as unidades tinham 200 (129 – 300) pacientes em terapia renal substitutiva.

Em relação aos aspectos considerados no atendimento nutricional de rotina, 99% dos nutricionistas analisam regularmente os exames laboratoriais e igual percentual avaliam o ganho de peso interdialítico, 95%, o apetite; 94%, os sinais e sintomas do trato gastrointestinal; 90% consideram o hábito alimentar; e 85%, as mudanças no peso seco.

O tipo e a periodicidade de uso de ferramentas de avaliação nutricional estão demonstrados na Figura 1. Dentre as seis ferramentas questionadas, as que apresentaram maior percentual de não utilização pelos participantes foram força de preensão manual (78%), bioimpedância (60%) e Malnutrition Inflammation Score – MIS (51%). As mais utilizadas, sendo com ou sem periodicidade estabelecida, foram inquéritos dietéticos (96%), Avaliação Global Subjetiva – AGS (83%) e composição corporal por antropometria (79%).

**Figura 1.** Utilização e frequência de ferramentas de avaliação nutricional.

Ao compararmos a utilização das ferramentas entre as unidades financiadas principalmente pelo sistema público e pelo privado, independentemente da periodicidade, encontramos diferença estatística apenas em relação à bioimpedância, sendo usada em 33% das unidades com financiamento público e em 64% daquelas com financiamento privado (P < 0,001).

A mediana (interquartis) do número de pacientes por hora mensal de trabalho do nutricionista foi 1,6 (1,0 – 2,3). Ao dividirmos em dois grupos de acordo com essa mediana, observamos que um percentual maior de nutricionistas com menos pacientes por carga horária utiliza as ferramentas de avaliação nutricional, independentemente da periodicidade, apesar da diferença ter sido pequena (Figura 2).

**Figura 2.** Utilização de ferramentas de avaliação nutricional, independentemente da periodicidade, de acordo com a mediana do número de pacientes da unidade por carga horária mensal do nutricionista.

As estratégias em casos de risco ou presença de desnutrição utilizadas com mais frequência (sempre ou quase sempre) pelos nutricionistas foram a orientação de incremento energético e proteico por meio de alimentos (97%) e o aumento da periodicidade das visitas (88%), seguidos pela prescrição de suplementos nutricionais industrializados (81%), discussão com a equipe multidisciplinar (80%) (Figura 3). A menos utilizada foi a prescrição de suplementos caseiros, sendo que 42% os prescrevem quase sempre ou sempre.

**Figura 3.** Estratégias apontadas pelos nutricionistas em casos de risco ou diagnóstico de desnutrição.

Com relação aos suplementos nutricionais industrializados orais, 7% (N = 14) das unidades os fornece a alguns pacientes que apresentam indicação; 10% (N = 20), a todos com indicação; e as demais não fornecem. Dentre as 34 unidades que fornecem suplemento, 31 são financiadas principalmente (N = 13) ou exclusivamente pelo SUS (N = 18).

Para a questão “Em casos de risco ou diagnóstico de desnutrição em que suplementação nutricional industrializada é indicada e o paciente não possui recursos financeiros para a aquisição, o serviço público de saúde fornece?”, as respostas foram: nunca, 17%; quase nunca, 27%; metade das vezes, 14%; a maioria das vezes, 27%; sempre, 6%; e não sabe, 9%. Dentre as regiões, a que fornece suplementação alimentar gratuita com maior frequência, entre metade das vezes a sempre quando solicitado, foi a região Sul (71%), seguida da Nordeste (55%), Norte (50%), Centro-Oeste (49%) e Sudeste (40%).

A Figura 4 mostra as repostas para a questão “Com base na sua prática clínica nesta unidade, o poder aquisitivo dos seus pacientes e a disponibilidade do serviço público de saúde, quando é necessária a indicação de um suplemento alimentar industrializado, com qual frequência você prescreve fórmulas padrão, fórmulas especializadas e módulos de nutrientes?”. A frequência de prescrição de fórmula padrão e especializada foi bastante semelhante e mais frequente do que a de módulos de nutrientes.

**Figura 4.** Frequência de prescrição de diferentes tipos de suplementos orais.

## Discussão

Neste trabalho, que contou com a participação de cerca de um quarto dos nutricionistas que atuam em unidades de diálise do Brasil, encontramos que a maior parte dos nutricionistas utiliza diversas ferramentas de avaliação nutricional e estratégias de intervenção em casos de risco ou diagnóstico de desnutrição, porém a frequência de utilização de tais ferramentas e estratégias apresentou bastante variabilidade.

No atendimento nutricional de rotina, a maioria dos nutricionistas afirmou analisar regularmente exames laboratoriais, ganho de peso interdialítico, apetite, sinais e sintomas do trato gastrintestinal, hábito alimentar e mudanças no peso seco, o que está de acordo com as práticas propostas pelas diretrizes de práticas clínicas de nutrição na doença renal crônica (KDOQI/AND)^1^, embora não tenha sido possível identificar a frequência com que essas análises são feitas.

As ferramentas de avaliação do estado nutricional mais empregadas foram antropometria, AGS e inquéritos dietéticos, possivelmente devido ao seu menor custo e maior facilidade de aplicação, sendo tais ferramentas recomendadas por fornecerem informações confiáveis a respeito da condição nutricional do paciente. Em relação à periodicidade, a diretriz KDOQI/AND sugere que a avaliação do estado nutricional seja abrangente e realizada pelo menos nos primeiros 90 dias do início da diálise, anualmente, ou quando indicado por triagem nutricional ou padronização do serviço^1^. Em artigo anterior com resultados referentes ao mesmo questionário, foi relatado que 64% dos pacientes são atendidos mensalmente pelos nutricionistas. Entretanto, de acordo com as informações levantadas, não foi possível identificar quais atividades especificamente são realizadas no atendimento mensal, ou seja, se somente aquelas baseadas em sintomas clínicos e exames de rotina ou se incluíam avaliação nutricional mais abrangente.

Com relação à avaliação da composição corporal dos pacientes, em 40% das unidades ela é realizada por meio da bioimpedância elétrica. Vale destacar que a maioria das unidades que têm disponível o equipamento de bioimpedância são aquelas mantidas com recursos privados, o que pode ser explicado pelo seu elevado custo, especialmente do modelo multifrequencial, sugerido pela diretriz KDOQI/AND como o mais indicado para essa população^1^. Outra ferramenta pouco utilizada foi o dinamômetro para aferição da força de preensão manual. Essa medida tem ganhado importância por sua relação com a massa muscular e com a funcionalidade e tem sido sugerida para ser empregada como o principal marcador para identificação de pacientes com sarcopenia^5^. Essa ferramenta também é indicada pelas diretrizes como marcador de estado nutricional e funcional^1^. Embora não avaliado, é possível supor que o seu pouco uso pelos nutricionistas seja também relacionado ao custo.

Nutricionistas com menor número de pacientes por hora mensal utilizam com mais frequência as ferramentas de avaliação nutricional, embora por uma pequena diferença em relação aos demais. De fato, uma pesquisa realizada com nutricionistas que atuam em unidades de diálise nos EUA, a elevada quantidade de pacientes por profissional foi apontada por 40% dos 951 participantes como uma barreira para implementação de condutas sugeridas por diretrizes^6^.

Cabe ressaltar que o tempo despendido em outras atividades nas unidades de diálise, como gestão de serviços de alimentação e atendimento de pacientes em tratamento conservador, não foi questionado, impossibilitando avaliar a influência dessa atuação em outras atividades nas características e qualidade do atendimento clínico ao paciente em diálise. Um trabalho norte-americano que incluiu 14 nutricionistas em um estudo de tempo e movimento encontrou que, em média, apenas 25% do tempo foi dedicado ao cuidado direto dos pacientes. Os cuidados indiretos, que incluem tempo despendido em planos de cuidados, checagem e anotações em prontuários, comunicação com outros profissionais, entre outros, ocuparam 56% do tempo^7^. Como evidenciado em outra investigação com a participação de 466 nutricionistas, atividades administrativas diminuem o tempo disponível de interação com os pacientes, tão necessário para melhorar os desfechos nutricionais^8^.

Uma das estratégias utilizadas por quase a totalidade dos nutricionistas, na presença de risco ou diagnóstico de desnutrição, foi o fornecimento de orientação dietética visando ao incremento de alimentos para maior aporte de energia e proteína. Essa é a recomendação indicada pelas diretrizes como abordagem inicial do cuidado nutricional. Caso isso não produza os efeitos desejados, a suplementação oral é então recomendada como uma estratégia válida. No presente trabalho, 81% dos nutricionistas referiram prescrever suplementos nutricionais industrializados para os pacientes nessa condição.

A suplementação oral pode fornecer um adicional energético de cerca de 7–10 Kcal/Kg/dia e proteico de 0,3–0,4 g/Kg/dia, sendo então necessária uma ingestão espontânea mínima de 20 Kcal/Kg e 0,4–0,8 g de proteína/kg a fim de atender às recomendações de aporte energético e proteico diárias^9^. Estudos mostram que o uso de suplemento nutricional, especialmente durante a sessão de hemodiálise, minimiza os efeitos catabólicos do procedimento, o que pode contribuir para promover melhora da condição nutricional. Em uma investigação com pacientes com evidências de desnutrição, a suplementação oral ao longo de seis meses levou ao aumento de 14% do score da AGS, além da elevação das concentrações de albumina e pré-albumina^10^. Embora haja evidências de benefícios com essa prática, os dados mostram que poucas clínicas (n = 34) fornecem suplementação aos seus pacientes e surpreendentemente 31 delas são financiadas parcialmente ou integralmente pelo SUS. Especulamos que tais clínicas possam estar vinculadas a unidades hospitalares, o que facilitaria a disponibilização de suplementos aos pacientes em tratamento dialítico.

Já em relação à participação do sistema público de saúde no fornecimento gratuito de suplementação aos pacientes com limitação financeira, observa-se discrepância entre as regiões do país, sendo a região Sul do país a mais bem amparada. Em contrapartida, a região Sudeste fornece suplementação gratuitamente a apenas 40% dos pacientes que têm indicação de uso.

Analisando os suplementos nutricionais específicos para indivíduos com doença renal crônica em diálise disponíveis em nosso país, percebe-se sua característica hipercalórica (≥1,5 Kcal/mL) e com elevado aporte de proteínas (>13 g/200 mL). Quando comparados a versões hipercalóricas e hiperproteicas padrão, apresentam menor teor de sódio e potássio, com teor de fósforo variável dependendo do produto. Por outro lado, ao compararmos o preço desses suplementos, os específicos custam cerca de 50% mais do que os suplementos padrão. Assim, na indicação do tipo de suplementação ao paciente (específico ou padrão), o nutricionista deve considerar individualmente aspectos clínicos e bioquímicos do paciente, bem como a viabilidade financeira para que ele possa manter o tratamento pelo período indicado, nas situações em que não haja fornecimento do suplemento pelo sistema público.

Como limitação, destacamos a possibilidade de que os achados não reflitam fielmente a realidade nacional, uma vez que a divulgação da pesquisa foi realizada por meio de aplicativo de mensagens de profissionais previamente organizados, além das redes sociais da SBN. Como pontos fortes, ressaltamos a originalidade e a participação de aproximadamente um quarto dos profissionais atuantes em unidades de diálise no país.

Com as informações levantadas neste trabalho, observamos a diversidade nas práticas de atuação do nutricionista em diálise no Brasil em relação à avaliação e intervenção nutricionais em casos de risco ou de desnutrição. Entre outras causas, essa diversidade reflete a falta de regulamentação da atuação desse profissional, com o estabelecimento de carga horária mínima baseada no número de pacientes e nas atribuições desempenhadas pelo profissional. O estabelecimento de parâmetros e de rotinas adequadas de avaliação e intervenção nutricionais é relevante para a melhoria da qualidade de atendimento e de vida dessa população.

## Material Suplementar

O seguinte material on-line está disponível para o presente artigo:

Questionário extraído do Google Forms: PERFIL E PRÁTICAS DO NUTRICIONISTA EM UNIDADES DE DIÁLISE BRASILEIRAS.
